# Understanding intracranial aneurysm sounds via high-fidelity fluid-structure-interaction modelling

**DOI:** 10.1038/s43856-023-00396-5

**Published:** 2023-11-09

**Authors:** David A. Bruneau, David A. Steinman, Kristian Valen-Sendstad

**Affiliations:** 1 Department of Mechanical & Industrial Engineering, University of Toronto, Toronto, ON Canada; 2 Department of Computational Physiology, Simula Research Laboratory, Oslo, Norway

**Keywords:** Aneurysm, Aneurysm

## Abstract

**Background:**

Since the 1960s, the origins of intracranial aneurysm bruits and musical murmurs have been debated, with proposed mechanisms ranging from self-excitation (i.e., resonance) by stable pulsatile flow, to vibration caused by unstable (laminar vortex shedding or turbulent) flow. This knowledge gap has impeded the use of intracranial sounds a marker of aneurysm remodelling or rupture risk. New computational techniques now allow us to model these phenomena.

**Methods:**

We performed high-fidelity fluid-structure interaction simulations capable of understanding the magnitude and mechanisms of such flow-induced vibrations, under pulsatile flow conditions. Six cases from a previous cohort were used.

**Results:**

In five cases, underlying flow instabilities present as broad-band, random vibrations, consistent with previously-described bruits, while the sac also exhibits resonance, rocking back and forth in different planes of motion, consistent with previously described musical murmurs. Both types of vibration have amplitudes in the range of 0.1 to 1 μm. The murmurs extend into diastole, after the underlying flow instability has dissipated, and do not exhibit the characteristic repeating frequency harmonics of previously hypothesized vortex-shedding mechanisms. The remaining case with stable pulsatile flow does not vibrate. Spectrograms of the simulated vibrations are consistent with previously reported microphone and Doppler ultrasound recordings.

**Conclusions:**

Our results provide a plausible explanation for distinct intracranial aneurysm sounds and characterize the mechanical environment of a vibrating aneurysm wall. Future work should aim to quantify the deleterious effects of these overlooked stimuli on the vascular wall, to determine which changes to the wall makeup are associated with vibration.

## Plain language summary

Brain aneurysms are weak areas of an artery in the brain that form a bulge. Most aneurysms do not rupture, but when they do, most patients die or are severely disabled. Unruptured aneurysms are often found by chance, but the risk of complications from treating them can be higher than the risk of rupture. Aneurysms are known to produce sounds, but the connection between the sounds and the resulting vibrations that may further weaken the artery wall are poorly understood. In this study, we modelled the possible vibration of aneurysm walls during turbulent blood flow. The frequency patterns of the vibrations were consistent with sound recordings obtained from patients. Our findings could enable potentially problematic aneurysms to be more easily identified in the future.

## Abstract (web summary)

Bruneau et al. simulate high-fidelity fluid-structure interactions to model intracranial sounds. Spectrograms of the simulated vibrations are consistent with previously-reported microphone and Doppler ultrasound recordings.

## Introduction

Intracranial aneurysms are prevalent (~1 in 30 adults) and with high mortality and morbidity if the aneurysm ruptures, but have a low annual rupture risk^1^. For unruptured aneurysms detected incidentally, there is a need for more reliable indicators of rupture risk beyond the basic systemic and morphological characteristics currently used^2^. Despite a large body of research into local hemodynamics as a proxy for wall integrity, there are no universally accepted hemodynamic indicators of rupture risk^3^. More recently, flow instability has been identified in some aneurysms via computational fluid dynamics (CFD)^4,5^, and it has been speculated that flow-induced wall vibration could be a mechanobiological stimulus for deleterious wall remodelling or rupture through a variety of possible mechanobiological pathways^6^.

From earlier studies, some intracranial aneurysms have been found to produce sounds, in the range of 100–800 Hz^7,8^; however, the source and mechanism of the sounds has been debated since. While some have speculated that pulsation can cause an aneurysm (or other types of lesion) with stable flow to self-excite^9–11^, most seem to believe vibrations are not caused by blood pulsation on its own^12,13^. Some have suggested that sounds could be caused by the aneurysm wall simply transmitting the frequency content of fluid vortex-shedding or “turbulence”^7,14^, while others have proposed that resonance of the aneurysm structure, in response to flow instability, is creating the sound^15^. If we assume resonance is taking place, the characteristic vibration mode shapes, and therefore the locations of greatest vibration, are not, however, clear from the literature. “Breathing” mode vibration (aka “fluid Helmholtz resonator”), consisting of uniform, radial expansion and contraction of the aneurysm sac^15^, has been considered as the only mode of vibration by most mathematical modelling studies^12,13,16^. To be tractable, however, such mathematical models must rely on idealized geometries, and simplify or ignore the internal fluid flow.

Recent computational and in vitro studies have identified additional modes of vibration where the whole sac is rocking back and forth in a particular direction^17,18^. While Ferguson^7^ and later Strother et al.^19^, postulated that aneurysm vibration causes additional stress which could lead to rupture, it is not currently possible to measure the magnitude of this vibration in vivo. Even if the vibrations are not sufficiently large to produce excessive stresses in the wall, it is possible that high-frequency wall stresses can lead to degenerative changes in mural cells, via damage to endothelial cells^20^ or through a reduction in active force of smooth muscle cells^21^. To better understand the scale of this mechanobiological stimulus and its importance, simulations can be used to estimate the prevalence, frequencies and magnitudes of these vibrations, while including a more realistic model of the vibration-inducing blood flow in the aneurysm sac.

Previous fluid-structure interaction (FSI) studies of aneurysms focused on low-frequency (~1–10 Hz) wall motions and stresses due to pressure pulsation^22,23^; however, these studies did not report vibrations, possibly due to their use of low temporal resolution and/or numerical schemes that are known to dissipate any flow instability that could drive such vibrations. We have previously performed proof-of-concept studies with high-fidelity FSI (i.e., high temporal resolution *and* a minimally dissipative numerical scheme^6^) capable of capturing high-frequency aneurysm wall motions in the hundreds of Hz (i.e., flow-induced vibration). Those studies innately incorporated realistic aneurysm geometries *and* realistic flow instabilities, addressing some of the limitations of previously mathematical modelling studies of aneurysm vibrations; however, they also only modelled flow under steady^6^ or ramped^17^ flow conditions, the latter focusing on the transition to flow instability. Thus, those studies presented physical, *but not physiologic*a*l*, vibrations in response to both vortex-shedding/harmonic and transitional/turbulent flow instability. Physiologically, pulsatile flows are more complex, as they have adverse pressure gradients after systole that can serve to destabilize flow, but also lower diastolic flow rates that promote relaminarization. Quantifying the frequency and magnitude of flow-induced vibrations using realistic flow conditions is necessary to understand the importance of any possible mechanobiological stimulus.

In this study we performed the first high-fidelity FSI simulations of vibrating intracranial aneurysms, accounting for physiologically pulsatile flow, to elucidate the mechanism of aneurysm wall vibrations, and how these might explain previously recorded bruits and musical murmurs. In cases with unstable flow, we show that the underlying flow instabilities present as random vibrations with broad-band frequency content, consistent with previously observed bruits. The sac also exhibits resonance, in one or more vibration modes with a defined pattern of motion and narrowband frequency, resembling previously observed murmurs. No vibrations were observed in the remaining case with stable pulsatile flow. Both the bruits and murmurs have similar amplitudes in various cases, ranging from 0.1 to 1 μm. These pulsatile simulations show the temporal evolution of the bruits and murmurs, and the power spectra and spectrograms resemble previously reported microphone and Doppler ultrasound recordings.

## Materials and methods

### Case selection

**Fig. 1:**
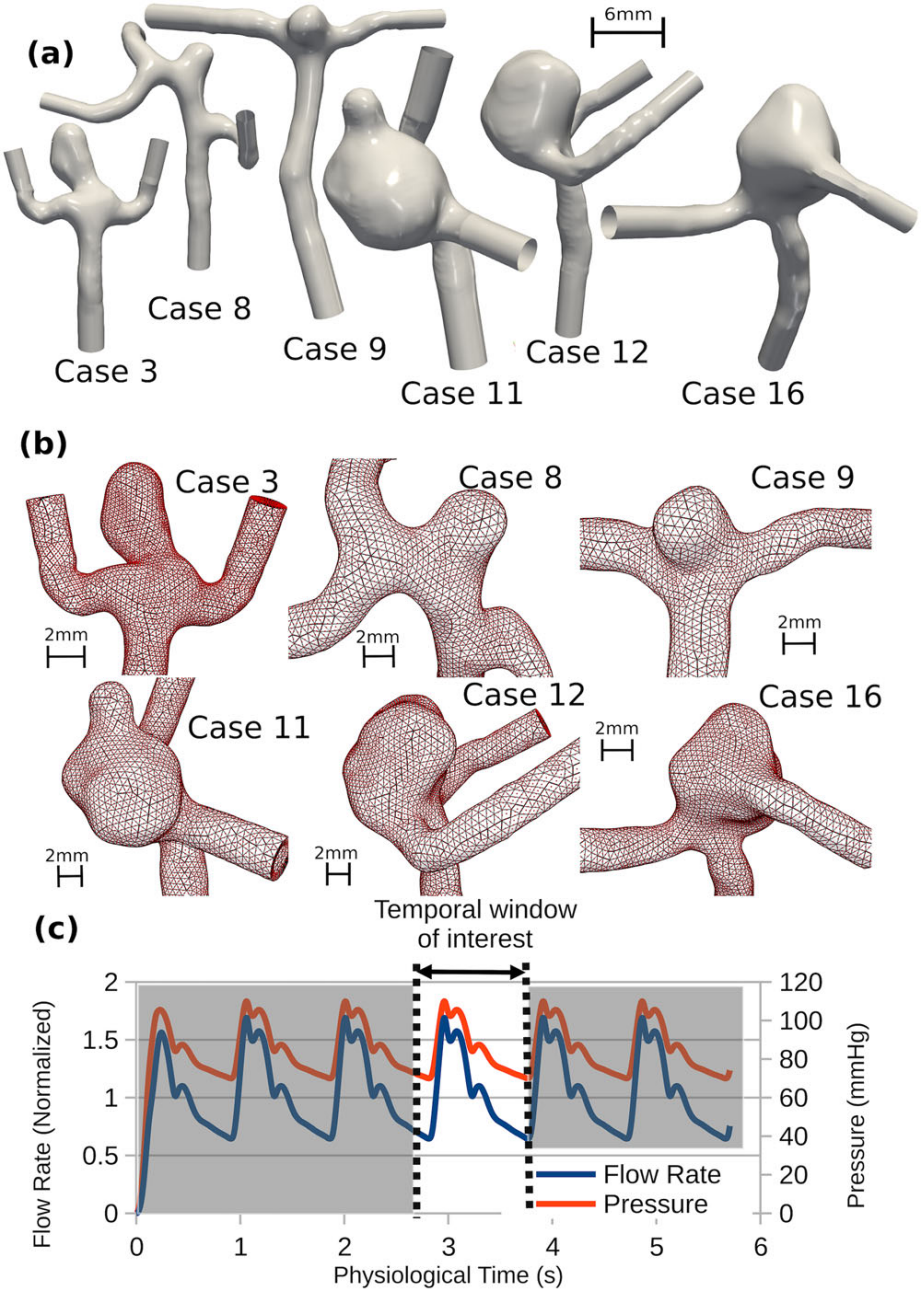
Model inputs and boundary conditions. **a** Input surface morphology (entire external geometry of the fluid region) for all aneurysm cases, on the same scale; the 6 mm scale bar applies to all models. **b** Meshes for all 6 cases. The surface triangular faces of the tetrahedral elements are outlined in black, with quadratic (mid-side) nodes superimposed in red. Note the black scale bar in each case, corresponding to 2 mm. **c** Imposed inflow rates and pressure over the 6 cycles simulated (5.6 s physiological time). The temporal window of interest identifies the fourth cycle from which later results are shown. Note the smooth increase in flow rate and pressure starting from 0 at *t* = 0.

Per Fig. 1a, b, we focused on six cases out of a cohort of 20 middle cerebral artery (MCA) aneurysms treated at the Department of Neurosurgery, University Hospital of North Norway from 2006 to 2008, which were previously characterized with CFD^24^. From the original cohort of 20, two were excluded because of inconclusive clinical data, and six could not be reliably segmented, leaving 12 aneurysms for CFD analysis. Of those cases, the five bifurcation aneurysms that had exhibited flow instability (Cases 3, 9, 11, 12, 16), and one sidewall aneurysm as a stable-flow control (Case 8) were included in the current study, noting that we have retained the original case numbers. Patient ages ranged from 30s-80s, and four of the six patients were female. Age and biological sex were not considered as covariates in this study, so only rupture status and bifurcation type are reported in Table 1.

**Table 1:** Computational and morphological details for each case.

Aneurysm case	3	8	9	11	12	16
Age group [years]	50–59	50–59	<50	60–69	80–89	70–79
Rupture status	Rup.	Unrup.	Rup.	Rup.	Rup.	Unrup.
Aneurysm type	Bifurc.	Sidewall	Bifurc.	Bifurc.	Bifurc.	Bifurc.
Sac volume [mm^3^]	43.4	12.1	15.2	339	275	297
Avg neck diameter [mm]	2.72	2.85	3.46	10.1	7.16	8.18
Sac height [mm]	5.82	2.58	2.55	7.77	6.33	6.08
Aspect ratio [–]	2.14	0.904	0.736	0.772	0.884	0.743
Mesh fluid volume [mm^3^]	166	141	209	922	498	546
Number of fluid elements	100,927	96,267	96,853	104,254	102,283	110,461
Number of solid elements	40,877	46,038	42,108	37,763	38,045	39,617
Average element size [mm]	0.238	0.234	0.261	0.371	0.317	0.329
Inlet flow rate [mL s^−1^]	1.61	1.11	1.92	3.25	1.66	2.12
Inlet Reynolds number [–]	254	224	284	369	258	308
Computation time [h]	59.2	31.9	57.0	77.3	82.2	94.5

Previous CFD and FSI verification studies have been performed to ensure that findings are relatively insensitive to spatial and temporal resolutions^24–26^. In the original study, the use of anonymized and de-identified patient images was approved by the University Hospital of North Norway’s committee for medical and healthcare research ethics, and patients provided informed consent. Further Research Ethics Board approval for our secondary use of this anonymous data was waived.

### Flow and pressure boundary conditions

Pulsatile flow was imposed by prescribing a cycle-average flow rate, and using it to scale a flow waveform shape derived from the internal carotid arteries (ICA) of older adults^27^, but damped by 10% to account for transit to the MCA^28^ (Fig. 1a). We assumed a mean cross-sectional average velocity of 0.37 m/s at the ICA inlet^24^, which implies that flow rate scales with inlet cross-sectional area. The internal pressure was assumed to vary between 80 and 120 mmHg^29^, from which was subtracted an intracranial pressure of 10 mmHg, which is within the typical range of 5–15 mmHg for a healthy adult^30^. The pressure waveform was assumed to follow the same waveform shape as the flow rate, scaled to minimum and maximum values of 70 and 110 mmHg (Fig. 1a). Internal pressurization was imposed as a distributed force on the inner wall of the solid geometry. To initialize the pulsatile flow and pressure, a sinusoidal segment starting at a value of zero and with zero slope, and ending with a value and slope matching the waveform was fitted to the pressure waveform and flow waveform for the first 0.25 s (Fig. 1c), to ensure smooth initial conditions.

At the inlet, fully developed (Womersley) velocity profiles were applied based on the prescribed flow rates. A zero-pressure (do-nothing) boundary condition was applied at the outlets. The deformable region of the simulation was defined by a sphere, placed at the centroid of the sac. The sphere radius was chosen to fully enclose the sac, with a minimum clearance of 0.5 mm, to encompass parent and daughter branch segments near the sac. The inlet and outlet arteries were otherwise rigid and fixed in space.

### Solid and fluid properties

A hyperelastic (St. Venant-Kirchoff) model was used for the wall material, with an elastic modulus of 1 × 10^6^ Pa and a Poisson’s ratio of 0.45, per previous FSI studies of intracranial aneurysms^6,22,23,31^. These parameters originated in Torii et al.^22^, where they were calibrated to match inflation experiments in a relevant pressure regime. Dynamic viscosity and density of the blood were assumed to be 0.0035 Pa s and 1000 kg m^−3^, respectively.

### Meshing

All six cases were meshed (Fig. 1b) so that the fluid domain contained roughly 100,000 tetrahedral elements, or an average element edge length of 0.234 to 0.371 mm depending on the case (Table 1). The fluid domain had two boundary layers of total thickness 0.25 mm. The fluid domain was truncated so that at least 10 inlet diameters length from the sac was considered, including ~3 diameters length of cylindrical flow extension to ensure circular inlets and outlets. The solid domain was meshed with 2 additional layers of elements, with a total thickness of 0.25 mm, corresponding to the average value of aneurysm wall thickness measured in aneurysm sacs resected from patients^32^, and consequently contained 35,000 to 45,000 elements.

Because medical image-based geometries are obtained in a pressurized state, whereas FSI simulations must be initialized in a state with no stress or internal pressure, we must infer a theoretical zero-pressure geometry. This was done following the approach described in our previous work^17^, namely, pressurizing each as-segmented geometry to the cycle-averaged pressure of 82 mmHg, and inverting those deformations to shrink the as-segmented geometry such that the pressurized computational model resembles the as-segmented geometry (see Supplementary Fig. 1 for more details).

### FSI solver

Computations were performed with turtleFSI^33^, a fully coupled, monolithic FSI solver, using a semi-implicit (shifted Crank-Nicholson) temporal discretization with *θ* = 0.501, and a Newton solver to iteratively solve non-linear parts of the monolithic formulation^34^. A timestep of 0.340 ms, or 2800 timesteps per 0.951 s cardiac cycle, was used, which has previously been shown to be sufficient for capturing flow instabilities with CFD^25^. The solution is second order accurate in time, and the use of P2P2P1 finite elements achieves third order accuracy in space, with an effective node spacing of 0.12 to 0.19 mm on average (half the average edge length of the mesh). As convergence criteria for the Newton iterations, the absolute tolerance was set to 10^−10^ and residual tolerance was set to 10^−9^, which were found to result in better cycle-to-cycle convergence with only a slight increase to the overall computational time. No turbulence model was included. Each simulation was run on 40 Intel Skylake cores of a Lenovo SD530 node of University of Toronto’s Niagara supercomputing cluster, with compute times ranging from 40 to 120 h (Table 1).

### FSI outputs and post-processing

A frequency of 25 Hz was used as the threshold for all high-pass and low-pass filters, since it encompasses 99% of the driving flow waveform power^26,35^. As such, for the wall, displacements above this 25 Hz threshold were considered “vibrations”, while those below were considered as “inflation” due to pressure pulsation. For the fluid, velocity fluctuations above 25 Hz were considered “flow instability”, while fluctuations below 25 Hz were classified as “stable flow”. To illustrate the level of flow instability, *Q*-criterion (a fluid mechanical parameter derived from the velocity gradient tensor, and used to highlight vortex cores^36^) of the high-pass and low-pass filtered fluid velocity was calculated per Natarajan et al.^37^. It is difficult to illustrate the scale of vibrations or flow instabilities by simply taking an instantaneous reading of the wall displacement or fluid velocity; similarly, it is challenging to create a spatial map of the locations of high and low vibration with a single temporal measurement. For this reason, to illustrate the overall scale and location of vibrations, we calculated the windowed Root-Mean Squared (RMS) amplitude of each of the *X*, *Y* and *Z* components of the high-pass filtered wall displacement (from here on, called “vibration amplitude”) and fluid velocity at every node (“flow instability amplitude”). A rectangular temporal window of 250 timesteps length was used. We then calculated the magnitude of the *X*, *Y* and *Z* components to create a single, representative value for each node, to create a spatial map of the locations of high vibration, and high flow instability. Finally, the 99^th^ percentile spatial value was plotted as a representative value for vibration and flow instability at a specific time, as 99th percentile values are considered less sensitive to spurious differences than maxima^38^.

Spectrograms were computed to illustrate the evolution of flow instability and vibration over the cardiac cycle, following methods and parameters originating in Natarajan et al.^35^ and Bruneau et al.^17^. Briefly, for the short-time Fourier transform, 7 windows were used for the temporal window of interest (1 cycle plus the preceding 0.1 s, total time of 1.051 s), with a window overlap of 75%, resulting in 1240 timesteps per window, a frequency resolution of 2.87 Hz and time resolution of 0.105 s. The units of the spectrogram were the input units squared, and the spectrograms were log-scaled. Spectrograms of each directional component, high-pass filtered above 25 Hz, were calculated at each node in the spherical deformable region of the simulation for both the velocity of the fluid nodes and the displacement of the wall nodes. The average of the nodal *X*, *Y* and *Z* point-wise spectrograms was taken as a representative, *X*, *Y* and *Z* directional spectrogram for the deformable region. The representative *X*, *Y* and *Z* directional spectrograms were then averaged to create the representative fluid velocity and wall displacement spectrograms. It is important to note that this differs from previous studies where spectrograms were computed from the *magnitudes* of vector quantities^17,35^ because, in vibration FSI simulations, the interface velocity changes direction often, and with sufficiently strong vibrations, this directional change would appear as an artifactual doubling of the modal vibration frequency owing to the magnitude being rectified at each zero-crossing.

Finally, vibration mode shapes were calculated by band-pass-filtering the bands in the displacement spectrograms that extended beyond the fluid velocity spectral content at those same frequencies. This band-pass filtered displacement was used to calculate the amplitude of vibration for the modes of vibration, by band-pass filtering the displacement to extract the 20–40 Hz wide bands on the displacement spectrograms. To extract the deformation pattern and amplitude of the bruit (i.e., the random wall vibrations not associated with the extracted modes), the same bands used to extract the modes were all *removed* from the wall displacement using a band-stop filter. Unless otherwise indicated, results are shown from the fourth cardiac cycle, which exhibited good cycle-to-cycle convergence in all cases, discussed in more detail in the Supplementary Note 1 and illustrated in Supplementary Fig. 2.

## Results

### Pulsatile flow behaviour

**Fig. 2:**
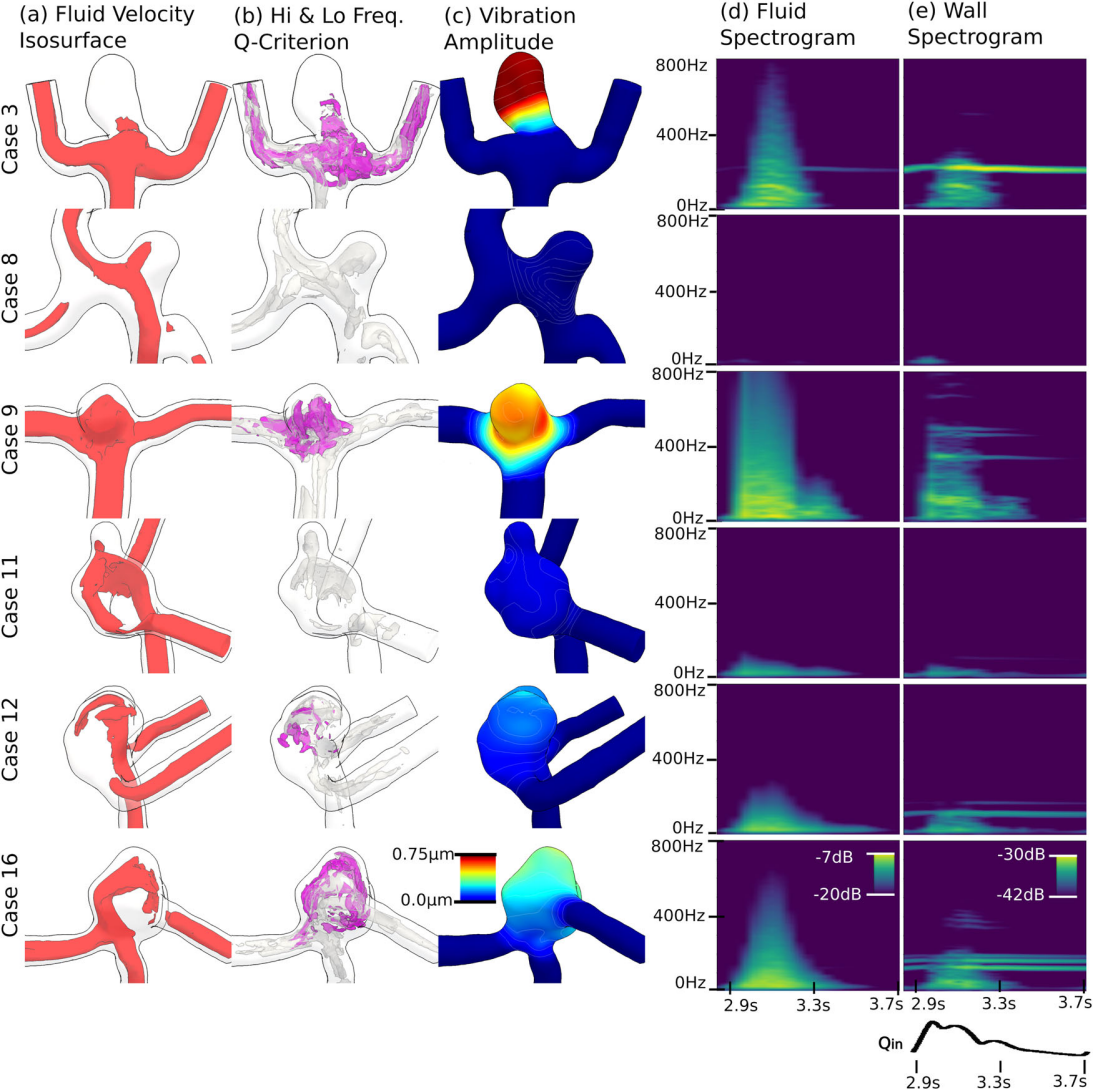
Location and frequency content of aneurysm flow patterns and vibrations. **a** Fluid velocity isosurfaces at 0.5 m s^−1^, shown in red. **b** *Q*-Criterion isosurfaces showing the extent of high-frequency flow instabilities in purple (*Q* = 5000) and (low frequency) stable vortex structures in light grey (*Q* = 50,000). **c** Vibration amplitude, with dark blue regions indicating no vibration and red indicating high vibration amplitude. **d** Fluid and **e** wall displacement spectrograms, where dark blue indicates low spectral power and yellow indicates high spectral power. For (**a**–**c**), results are shown at the time of maximum flow instability for each case, between 3.0 and 3.1 s physiological time. Case 8 had no flow instability and is shown at 3.1 s.

Qualitatively, the gross flow patterns shown by the velocity isosurfaces of the six cases (Fig. 2) were similar to those from previous CFD studies of the same cases^4,24^. The cases with flow instability in the current study (3, 9, 11, 12 and 16) were all unstable in previous studies, although Case 11 had only weakly unstable flow in the current study, likely due to the more realistic increased size of the neck during systole in the FSI simulations compared to previous (rigid-wall) CFD.

Cases with flow instability exhibited vibrations, while the stable flow control (Case 8) only exhibited very low levels of self-excitation at low frequencies (25-35 Hz) under *stable* pulsatile flow (Fig. 2). At the time of maximum flow instability, in most vibrating cases (3, 11, 12 and 16) the vibration amplitude was the greatest at the extreme top (dome) of the sac, while in Case 9, the vibration amplitude was largest on lateral side of the sac. In all but Case 8, the fluid spectrograms exhibited some degree of transitional or turbulent flow, as evidenced by the broad-band power spectra spread across frequencies >25 Hz at peak systole, which subsequently dissipated in diastole. Only Cases 3 and 9 exhibited noticeable regularly repeating bands in the fluid spectra, suggestive of harmonic (vortex-shedding) phenomena^39^. On the other hand, the displacement spectrograms for cases with flow instability (3, 9, 11, 12 and 16) exhibited irregularly repeating (i.e., non-harmonic) narrow bands in the hundreds of Hz that extended into diastole, after the dissipation of flow instability. Case 3 had a single band that extended through the cardiac cycle at 220 Hz; Case 9 had one strong band at 355 Hz and two weaker bands at 470 and 495 Hz; Case 11 had a very weak band at 110 Hz, and Cases 12 and 16 had two and three such bands, all in the 100-200 Hz range. To better illustrate the flow instability and wall vibration, we have included a real-time visualization of the Cases 3, 9, 12 and 16 (Supplementary Videos 1–4), which also include direct sonifications of the fluid and wall responses.

### Mode shapes and bruits

**Fig. 3:**
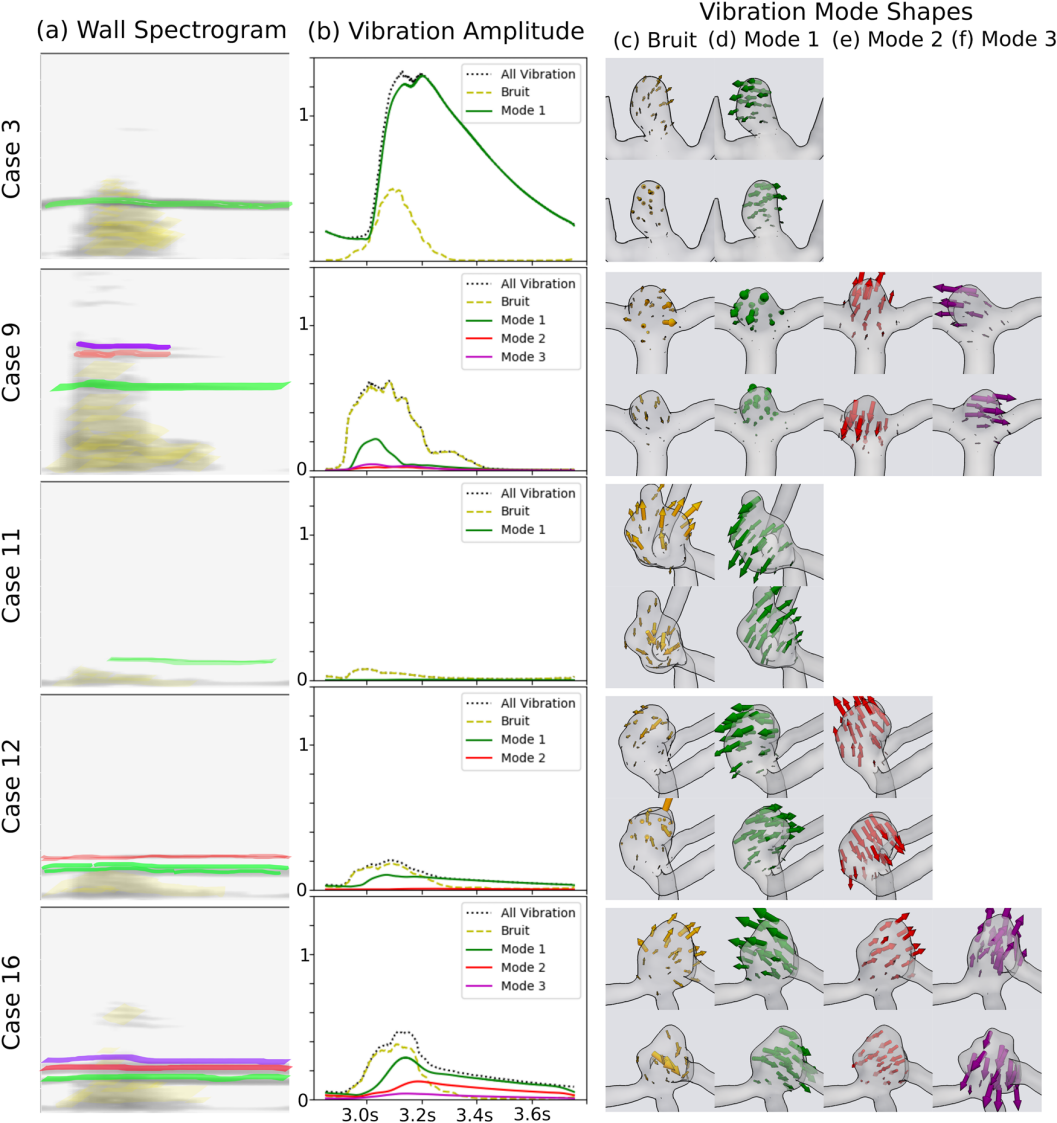
Summary of aneurysm vibration modal frequencies, amplitudes and mode shapes. **a** Spectrogram of wall displacement, with the bruit shaded in yellow, and rocking modes of ascending frequency shaded in green, red and purple. Subsequent panels of this figure follow the same colouring scheme. **b** Spatial 99th percentile value of the RMS vibration amplitude, in μm. The total vibration amplitude is displayed with a black dotted line; the rocking modes displayed in green, red and purple solid lines; and the remaining bruit displayed by yellow dashed line. **c** Vibration motion of the bruit, containing both expansion/contraction and random, rippling motions. **d**–**f** Mode shapes for **d** Mode 1, **e** Mode 2 and **f** Mode 3. The bruit motion and mode shapes are not to scale and are normalized to the size of the sac so that each mode of a specific case is shown at the same visual amplitude.

**Fig. 4:**
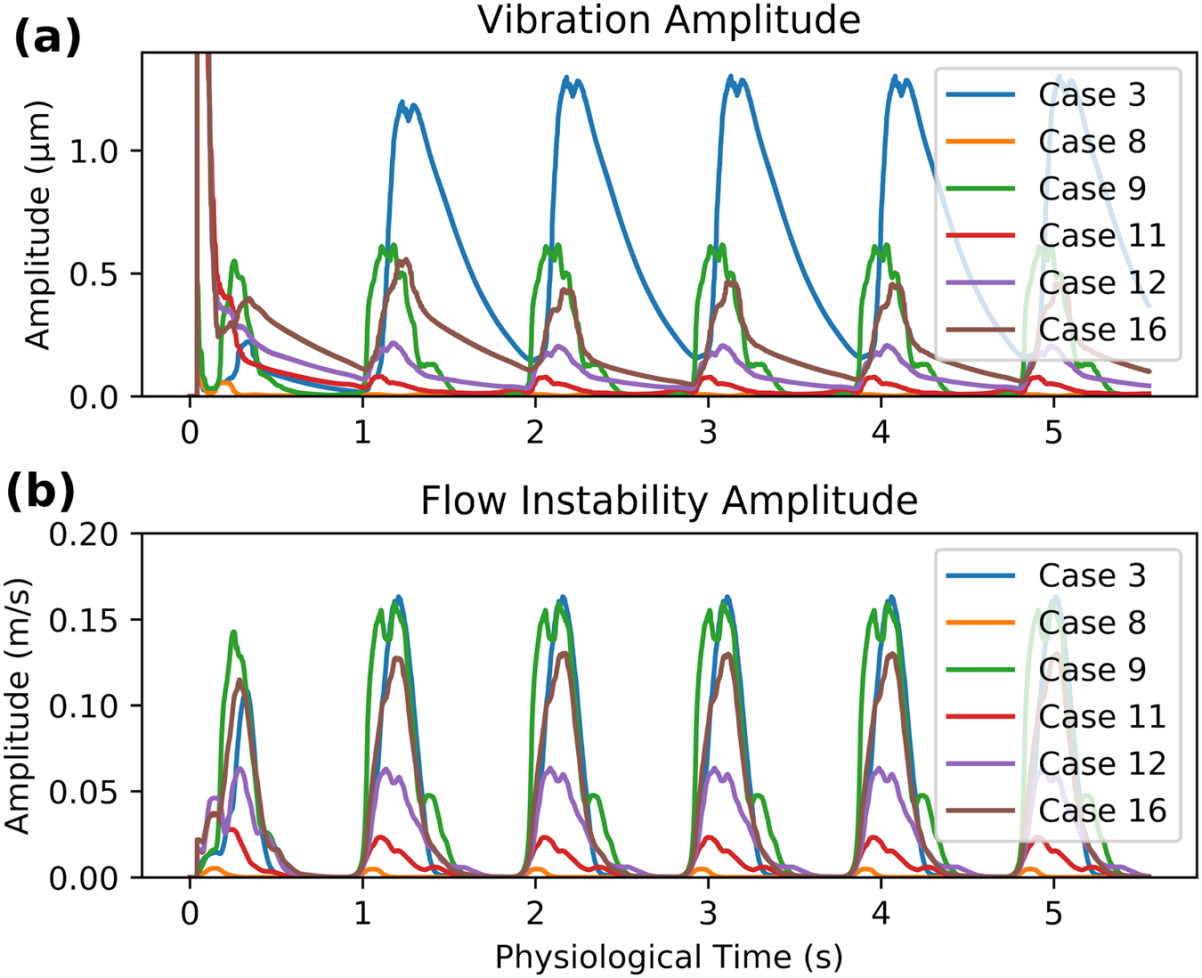
Cyclical and by-case variation in vibration and flow instability amplitude. Spatial 99th percentile value of the **a** Root-Mean-Squared vibration amplitude and **b** fluid velocity amplitude over the multiple cardiac cycles simulated.

For the bands in the displacement spectrograms that extended into diastole, band-pass filtering of the wall displacement vector revealed that each of these bands corresponded to structural rocking modes of the entire aneurysm sac, which were numbered by ascending frequency. Per Fig. 3, the shape of Mode 1 was similar in all cases: a rocking motion between the two outlet branches. Modes 2 and 3 consisted of similar rocking motions along a different axis (such as Cases 9 and 16, mode 2), or a “bouncing” motion of the entire sac (as in Case 12, mode 2 or Case 16, mode 3). Modal frequencies were related to the size of the aneurysm, with the largest aneurysms exhibiting the lowest frequencies (120–190 Hz in Case 16) and the smallest aneurysm exhibiting the highest frequencies (360–495 Hz in Case 9). The lowest frequency mode exhibited the highest vibration amplitude in all the cases with vibration (Fig. 3). The transitional or turbulent bruit transmitted by the wall, obtained by removing the rocking modes from the wall displacement, was a mixture of seemingly random rippling motion of the wall, mixed with expansion and contraction of the aneurysm sac with the centre of the sac staying relatively stationary in time. The amplitudes of the bruit and the rocking modes were comparable: in Case 3, the rocking mode had a larger amplitude (1.3 μm) than the bruit (0.5 μm), while in Case 16, the bruit had a slightly higher amplitude (0.4 μm) than the first rocking mode (0.3 μm) and in Case 9, 11, 12, the bruit had a considerably higher amplitude than each of the rocking modes. To better illustrate the difference between the repetitive nature of the mode shapes and randomness of the turbulent bruits, we direct the reader to the Supplementary Videos 5–8. Overall, vibration amplitudes had a clear ranking, with Case 3 vibrating the most (1.3 μm), then Cases 9 (0.6 μm) and 16 (0.5 μm), with Cases 12, 11 and 8 vibrating the least (Fig. 4a). The cases with the highest amplitude of flow instability exhibited the highest level of vibration, while those with no flow instability vibrated the least (Fig. 4b).

### Overall scale of vibrations

**Fig. 5:**
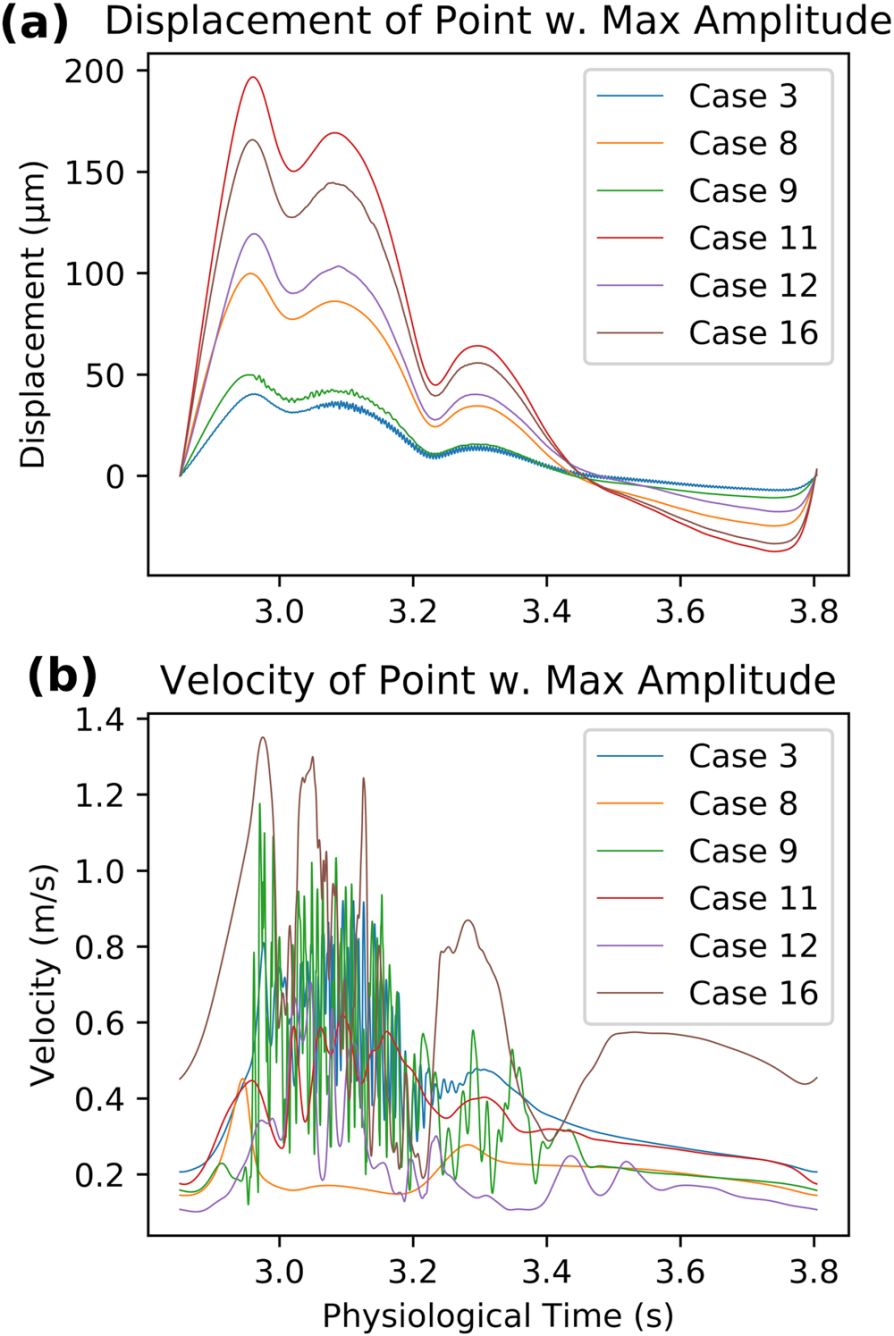
Overall scale of aneurysm vibrations and flow instabilities. Point traces of **a** wall displacement and **b** fluid velocity magnitude at the point with maximum vibration amplitude and flow instability amplitude, respectively.

The overall scale of the wall vibration was small relative to displacement induced by pulsation of intramural pressure (Fig. 5a). In the displacement point traces of cases with no/low flow instability (Case 8 and 11), the self-excited vibrations are not visible. Vibrations are visible in Case 3, 9, 12 and 16, but at most, in Case 3, the vibration RMS amplitude reaches only ~4% of the overall wall displacement, likely because the pulse pressure variation was much larger than the high-frequency pressure fluctuations due to flow instability. In contrast, the fluid velocity trace is dominated by flow instability (Fig. 5b).

### Dependence of vibration on model parameters

**Fig. 6:**
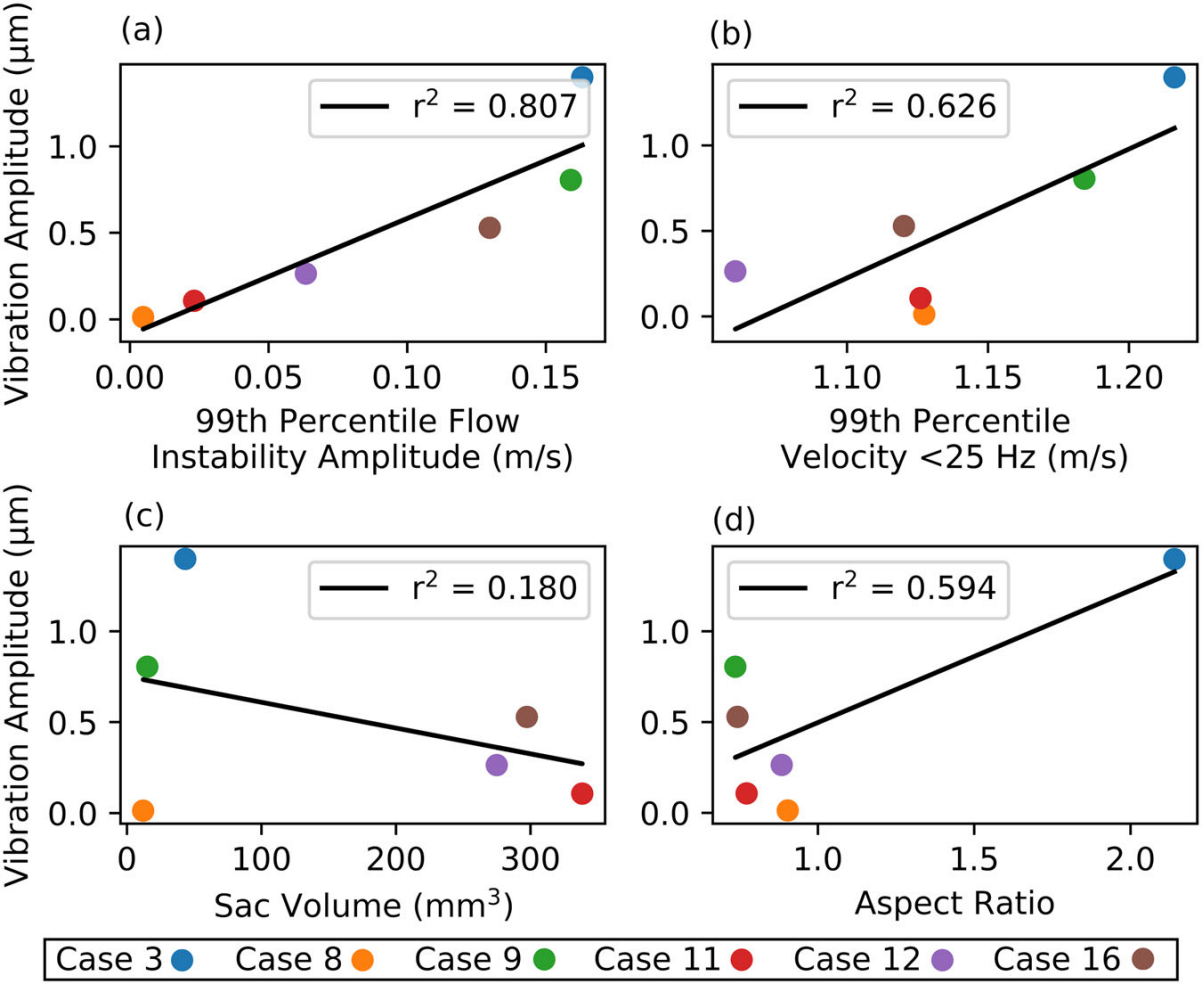
Dependence of aneurysm vibration on geometric and blood flow parameters. Dependence of 99th percentile vibration amplitude on **a** 99th percentile flow instability amplitude, **b** 99th percentile, fluid velocity <25 Hz, **c** aneurysm volume and **d** aspect ratio. *n* = 6 independent simulations, with data points following same by-case colour scheme as Figs. 4 and 5.

Univariate linear regressions showed that the parameter most predictive of vibration was 99th percentile flow instability amplitude (Fig. 6), with a goodness-of-fit of 0.81. Maximum fluid velocity in the model (<25 Hz), reflecting bulk (stable flow) velocities was more weakly predictive at 0.64. Of the two morphological predictors, aspect ratio was loosely correlated with vibration amplitude, while sac volume was not.

## Discussion

Since the 1950s, it has been known that some intracranial aneurysms produce sounds, some of which were described as having a “musical” quality^7^. The studies that recorded these sounds could only speculate about their causes, ranging from self-excitation of the aneurysm^9^, to the wall transmitting the sound of “turbulence”^7^ or “vortices”^40^, and the possibility that the wall is resonating at a structural natural frequency, adding its own signature on top of the fluid turbulence or vortex shedding^41^. Since the late 1990s, there have been analytical studies that investigated aneurysm resonance considering simplified spherical aneurysms but also presuming the mode shape of the vibration as part of the model setup, usually radial or breathing mode vibration^12,13^.

In the present study, we performed pulsatile FSI simulations of wall vibration in a cohort of aneurysms. By extracting the dominant modes of vibration, our results show that aneurysm vibrations (i) result predominantly from flow instability and not flow pulsation, and (ii) consist of *both* transmission of the fluid turbulent bruit and oscillatory rocking modes of the aneurysm sac. We found that the bruits presented as a broad-band instability (in some cases displaying weak, repeating harmonic structures) spread from 25 Hz into the hundreds of Hz, while the rocking modes presented as narrow bands over 100 Hz with a frequency that decayed slightly with intramural pressure, extending into diastole. That these bands extend late into diastole is likely due to the lack of external cerebrospinal fluid or viscoelastic damping in the current study. While these bands might dissipate earlier if those sources of damping were added, they would likely not be eliminated, as David and Humphrey^42^ have demonstrated that even with cerebrospinal fluid and a viscoelastic damping model, the aneurysm oscillation can persist for multiple cardiac cycles before subsiding.

The displacements resulting from the bruits and rocking modes were of a *similar* scale, with amplitudes in the ~0.1–1.3 μm range. In previous (rigid-wall) CFD studies^35,39^, fluid vortex-shedding presented as *repeating* narrowband harmonics in fluid spectrograms, typically starting in the lower frequency (25–100 Hz) range and decreasing in lock-step with flow rate^39^. Unlike spectra of narrowband flow instability, the wall displacement spectra of the rocking modes in the present study do not consist of strong repeated bands (i.e., few or no harmonics in the spectrum), but rather they produce a nearly pure tone at a specific frequency. As such, our results suggest that axisymmetric rocking modes of the sac are the cause of the musical murmurs (also known as spikes) recorded in previous studies^8,15,40,43^, while the underlying flow instability is the cause of previously recorded bruits. As previously recorded murmurs did not typically exhibit a strong repeating structure (i.e., harmonics), our results suggest that most previously recorded murmurs do not reflect fluid vortex-shedding phenomena. Olinger and Wasserman^41^ suggested that the mechanism of aneurysm vibration could be due to a fluid Helmholtz resonator (aka breathing mode vibration), where the sac of the aneurysm resonates with a specific vibration frequency in response to either vortex shedding or “turbulence” over a range of frequencies, and that the bruits and frequency spikes in the aneurysm sound recordings represent fluid turbulence and aneurysm vibration, respectively. Of the previously proposed explanations for aneurysm sounds, this theory best describes what was observed in the present study. In our study, aneurysms with *stable* or weakly unstable flow exhibited very weak, low frequency (25–40 Hz) self-excited vibration when the changes in pressure in the driving flow waveform were the sharpest, suggesting that this is not the primary driver of aneurysm vibration.

Interestingly, the breathing mode vibration described by Olinger and Wasserman^41^ and in several previous studies^12,13^ was not the main source of narrow-band vibration in our study. Some radial expansion and contraction did comprise part of the bruit in the current study, but it did not occur with regularity in any of the cases, and was usually coupled with a folding motion of the sac due to the irregular geometry of the sacs in all cases—remember, wall thicknesses and material properties were assumed to be uniform in the present study—usually folding in regions of sac concavity. Any narrowband content in the wall spectrogram that was not a rocking mode was also present in the fluid spectrogram, suggesting that the wall transmits both fluid vortex shedding and transitional/turbulent flow in the form of localized, random vibrations mixed with folding or breathing motions. In a previous study by the authors^17^, Case 9 exhibited what was identified as “folding mode” vibration which followed the frequency of vortex shedding in the fluid spectrum, suggesting that these modes of vibration do exist in aneurysms, but the mode shapes and frequencies are more variable than the rocking modes in response to different internal pressures and frequency, and are thus spread over a wider range of frequencies. These breathing mode vibrations require the aneurysm to push blood in and out of the sac, which is an additional source of damping, compared to the rocking mode vibrations, where the sac remains relatively constant in volume. The lack of a narrow-band breathing mode in the current study contrasts with nearly all previous analytical modelling studies of aneurysm vibrations, such as Shah and Humphrey^13^, where it was assumed that the main mode of vibration was radial expansion/contraction. Those studies used simplified geometries, whereas the current study highlights the importance of using realistic aneurysm geometry, as none of the aneurysm cases exhibited the same mode shapes as the previously assumed spherical aneurysm models.

**Fig. 7:**
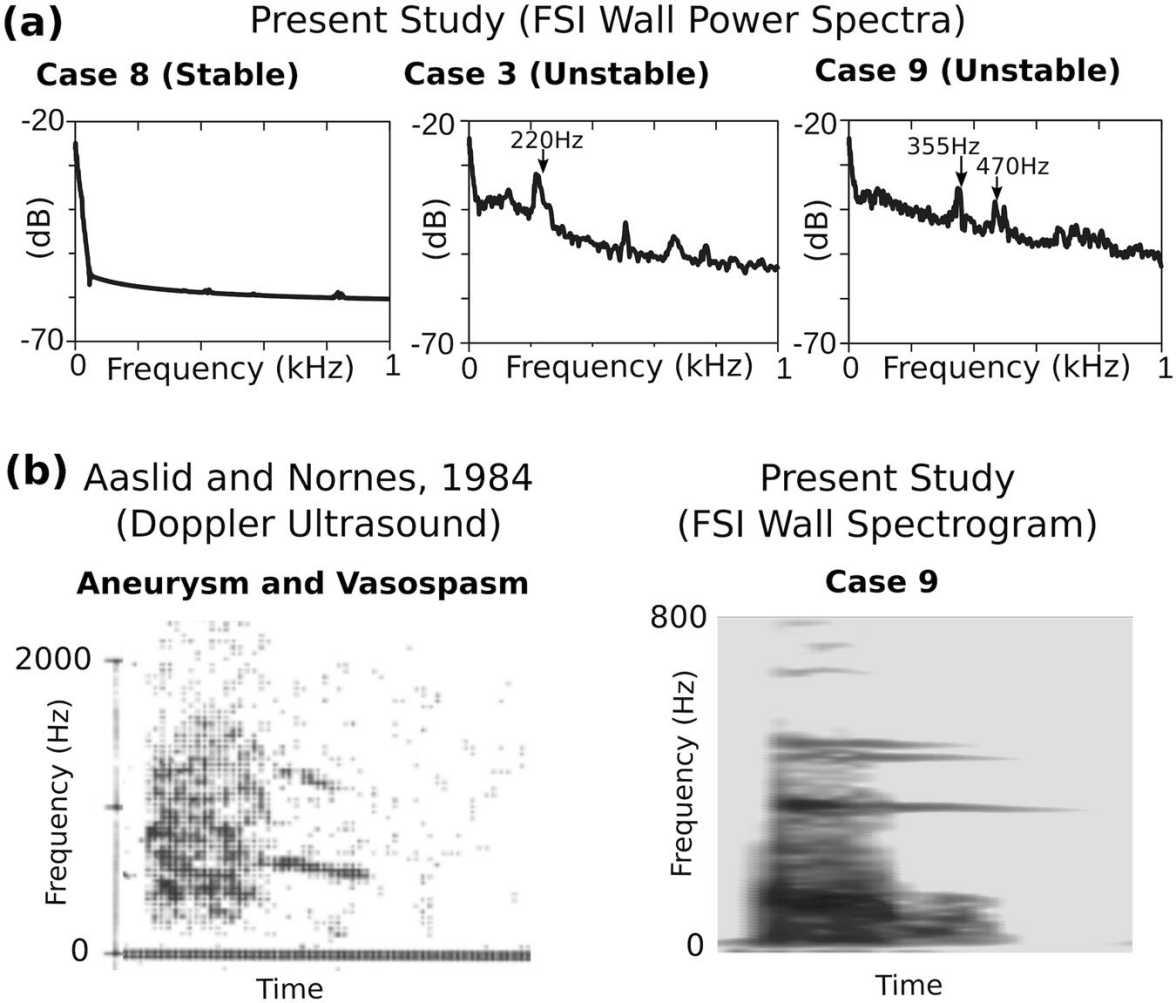
FSI models explain aneurysm vibrations consisting of both bruits and musical murmurs. **a** Power spectra of the wall displacement from selected cases, which resemble normal, single peak and double peak spectra previously recorded in vivo by Kurokawa et al.^8^, and shown in their Figs. 3, 4 and 6, respectively. **b** Comparison of Doppler ultrasound recording of aneurysm with vasospasm from Aaslid and Nornes^40^ and wall displacement spectrogram from Case 9 in the current study, with both showing bruit signals during systole followed by musical murmurs into diastole. (Panel from Aaslid and Nornes is protected by Copyright, is owned by The JNS Publishing Group, and is used with permission only within this document. Permission to use it otherwise must be secured from The JNS Publishing Group. Full text of the article containing the original figure is available at the jns.org.).

In our study, the spectra of computational aneurysm vibrations resembled spectra from clinical recordings of blood flow sounds. In contrast with our previous studies using steady or simplified flow conditions^6,17^, these pulsatile simulations show the temporal evolution of the bruits and musical murmurs, illustrating how they arise and subside over the cardiac cycle as, respectively, the flow destabilizes and then relaminarizes. Since the whole cardiac cycle is captured in power spectra in the present study, the power spectra and spectrograms from these simulations can now be more reliably compared to previous sound recordings from patients, while previous steady flow simulations only represent the flow and vibrations at peak systole^6^. The wall spectra in the current study bear resemblance to the variety of spectra observed in previous sound recordings of vessel wall abnormalities (aneurysms, stenoses, vasospasm, arteriovenous malformations). In Kurokawa et al.^8^, sonic recordings at the eyes of patients were found to exhibit a variety of spectral phenomena, including (i) a normal spectrum like a patient with no aneurysm (their Fig. 3), (ii) a single sharp peak (their Fig. 4), and (iii) multiple peaks (their Fig. 6), all in the range of 100–800 Hz. As shown in Fig. 7a, our FSI simulations exhibited comparable trends, as there were aneurysms with little sound (Case 11) to no sound (Case 8), a single strong peak (Case 3), or multiple peaks (Cases 9, 12, and 16). In the current study, the frequencies were in the same range (100–500 Hz) as in Kurokawa et al.^8^, but tended to be lower in frequency on average, especially in the larger aneurysms. In contrast to our findings, in that study there was no correlation between aneurysm size and frequency. We suspect that modelling aneurysmal contact with its surroundings would likely constrain the larger aneurysms and increase the effective stiffness and modal frequencies; this is analogous to a fixed-fixed beam, where the addition of one constraint results in much higher modal frequencies than a cantilever beam. The FSI models exhibited sharper peaks than the clinical recordings; this could be explained by the transmission of vibration through other tissues (eye, cerebrospinal fluid, etc.) in the clinical recordings, which would likely make the frequency peaks broader and less pronounced.

While there are no known studies that show the variation of frequency content over the cardiac cycle for an isolated aneurysm, Aaslid and Nornes^40^ presented Doppler ultrasound recordings of aneurysms with vasospasm. In one particular case, the recording had a strong band, which they called a musical murmur, at ~550 Hz superimposed over a systolic bruit ranging from 100 to 1500 Hz, with the band extending past the bruit into diastole (Fig. 7b). This recording resembles the spectrograms in the present study (Fig. 7b), which also have bands in the hundreds of Hz that extend into diastole beyond the underlying bruit. In Aaslid and Nornes^40^, the band dissipates in the middle of diastole, while in the current study, the bands extended longer into diastole due to the lack of external damping, as noted earlier. Again, the frequencies of the bands in the present study were lower than those in Aaslid and Nornes, and notably, the bands were less sloped with time. However, the slope of the bands in FSI simulations should depend on the wall thickness and pressurization, e.g., in preliminary parametric studies, we have noted that the bands can be more sloped if the effective stiffness of the sac is lowered. Aaslid and Nornes postulated that the strong correlation of the murmur frequency to the flow velocity indicated that the musical murmurs were caused by a vortex shedding phenomenon. However, past CFD studies have typically shown that harmonic vortex shedding presents as strongly repeated banding structures starting in the lower frequency (25–100 Hz) range^35,39^. Aaslid and Nornes noted that a harmonic of the band in their recording (Fig. 7b) was an artefact of their ultrasonic demodulation process. Our results suggest that single, narrow bands extending into diastole are modes of the vessel wall structure. As seen in the present study, in diastole these bands also vary in frequency, not due to the proportionality to flow velocity directly, but due to the change in internal fluid pressure, which stiffens the structure during systole and increases the modall frequencies.

Overall, the trends in vibrations among the six cases were consistent with theoretical mechanics of vibrations. Vibrations were strongest in the case with the highest amplitude of flow instability (Case 3) and less strong in cases with little/no flow instability (Cases 8 and 11), as indicated by the good correlation (*r*^2^ = 0.81) between flow instability amplitude and vibration amplitude. Case 3 also had visible bands in the fluid spectra, which could contribute to the high propensity to vibrate in that case. Structural properties of the aneurysms, such as the sac stiffness and mass due to their shape, influence vibrations; for example, Case 3 may have a high propensity to vibrate because of the narrow neck and high aspect ratio (which was found to be loosely correlated with vibration amplitude, *r*^2^ = 0.59), and is thought to be less resistant to motion due to a lower bending moment resistance about the neck. Aneurysms with a high aspect ratio are also known to be more prone to rupture^3^, thought to be due to fluid mechanical (lid-driven cavity) vortex and flow stagnation phenomena^44^; a larger cohort study on vibrations could reveal whether vibration is an underlying contributor for this risk factor. For Cases 9 and 16, the mode shapes and frequencies of the rocking modes were the same as in our previous ramped-flow study^17^, confirming that these modes are a structural property of the sac geometry.

We suspect that aneurysm vibrations have a mechanobiological effect on the wall. In 1970, Ferguson asserted that aneurysm vibration produces degenerative changes in tissue similar to the phenomenon of structural fatigue. In the current study, the magnitude of vibration is unlikely to add sufficient additional stresses to damage the wall by overloading, and we now know that human tissue does not experience structural fatigue in the same way as, say, metals; rather, vascular walls adapt positively or negatively to abnormal stimuli. Previous studies have shown a variety of pathways by which vibration can cause abnormal behaviour in blood vessels and their comprising mural cells. On a macro-scale, Roach^45^ found post-stenotic dilatation formed in response to bruits, and later showed that this occurred in response to vibrations at specific frequencies in resected human iliac arteries^46^. In another example, external vibration (>30 Hz) is widely known to cause vasoconstriction in the fingers (Hand–Arm Vibration syndrome), and higher frequencies result in greater reductions to blood flow^47^. While the mechanisms by which vibrations cause permanent vasoconstriction is not certain, Reda et al.^48^ proposed a cascade of events. First, exposure to vibration causes an acute reduction in blood flow, and subsequently, reduced wall shear stress exerted on the endothelium, which, in turn, causes chronic remodelling after long-term exposure to vibrations. Similar endothelial cell dysfunction can occur in vibrated tissues due to snoring^20^. However, most ruptured aneurysms, and even some unruptured aneurysms, do not have an intact endothelium^49^, so wall shear stress may not play the same role as in normal physiological remodelling. As such, another mechanism by which vibration could cause wall remodelling is by directly disrupting smooth muscle cell activity. In isolated cultures of smooth muscle cells, Bittle^50^ found proliferation of these cells when subjected to vibrations at 45 Hz. Further, Ljung and Sivertsson^21^ described a phenomenon where vibration caused a drastic reduction of the active force applied by smooth muscle cells, resulting in the passive structures bearing more load and potentially becoming damaged. The current study has described the mechanical environment of a number of vibrating aneurysm cases. Being that mural cell responses to high-frequency mechanical stimuli are largely unknown and under-studied, our data can be used to guide in-vitro experiments (amplitude and frequencies of vibration) on mural cells to better understand possible deleterious cell remodelling and hence the possible role of wall vibrations in aneurysm rupture risk.

### Limitations

Our aneurysm models assumed uniform wall thickness and near-linear material properties, and with no viscoelasticity or surrounding fluid or structures. Aneurysm walls are known to be non-uniform in both thickness and material properties, which will of course influence the specifics of the mode shapes and localized transmission of bruits, e.g., thinner regions will be more deformable. Aneurysms are often in contact with brain tissue or bone^51^; such contacts will drive up the modal frequencies of the model, but the exact contacts are not well known for the current aneurysm cases. Recent optical coherence tomography (OCT) studies have also highlighted the presence of subarachnoid trabeculae^52^, the distribution and mechanical properties of which are unclear. Preliminary results using a more nonlinear material model fitting tensile test data from Robertson et al.^32^ were found to result in altered vibration frequencies but with similar vibration phenomena, while inclusion of damping from cerebrospinal fluid served to reduce the duration of vibration of the rocking modes during the cardiac cycle, more consistent with the in vivo recordings shown in Fig. 7b. The overall phenomena, however, did not change. We also assumed generalized (but physiologically plausible) pulsatile flow rate and pressure conditions. It is important to note, however, that the aim of this study was not to simulate aneurysm wall vibrations in a patient-specific manner, but rather to reveal under replicable conditions, the phenomenology of flow-induced aneurysm wall vibrations when complex, pulsatile flow is modelled directly, rather than simplified to a steady flow or even an idealized forcing function as in previous studies.

### Future perspectives for FSI modelling

Preclinical models of aneurysms might allow for direct correlations and validations of FSI predictions, as well as helping to make the link between vibration phenomena and wall pathology. For clinical imaging, it is already possible to acquire at least some of the anatomic constraints (if not necessarily their material properties) from routine CT or MR imaging. From basic vibration theory, we can anticipate that such contacts will drive up the modal frequencies of the model. High field (7 T) MRI shows some promise for resolving aneurysm wall thickness and enhancement^53^ (the latter a possible proxy for wall composition and hence material properties), and OCT may one day even allow for individual mapping of arachnoid trabeculations^52^.

### Supplementary information

Supplementary Information

Description of Additional Supplementary Files

Supplementary Data 1

Supplementary Data 2

Supplementary Data 3

Supplementary Data 4

Supplementary Data 5

Supplementary Data 6

Supplementary Data 7

Supplementary Video 1

Supplementary Video 2

Supplementary Video 3

Supplementary Video 4

Supplementary Video 5

Supplementary Video 6

Supplementary Video 7

Supplementary Video 8

## Data Availability

FSI datasets are cumbersome to share; however, requests for subsets of data may be granted by reaching out to the corresponding author. Source data for the figures can be found in Supplementary Data 1–7.

## Code availability

The open-source turtleFSI solver is available at https://github.com/KVSlab/turtleFSI. The specific version we used^33^ is available at 10.5281/zenodo.10001935. Post-processing scripts are available upon request from the corresponding author.
