# Supplementary material for: Understanding intracranial aneurysm sounds via high-fidelity fluid-structure-interaction modelling
Source: Commun Med (Lond). 2023 Nov 9;3:163. doi: 10.1038/s43856-023-00396-5 (PMC10636010; doi:10.1038/s43856-023-00396-5)
Supplement: Supplementary file 1 — Supplementary Information [file 43856_2023_396_MOESM1_ESM.pdf]

Understanding Intracranial Aneurysm Sounds via High-Fidelity Fluid-Structure-Interaction Modelling

David A. Bruneau, David A. Steinman, Kristian Valen-Sendstad

Suppementary Note 1 – Cycle-to-Cycle Convergence

The L2 norm between each cycle (2 to 6) and the previous cycle (1 to 5) was calculated, as in [Ford et al., 2008], to illustrate cycle-to-cycle convergence (Supplementary Figure 2. This value was normalized to the difference between cycle 1 at 0.0 s and cycle 2 at 0.951 s. The L2 norm shows that the steepest convergence occurred in the first part of the second cardiac cycle for all cases, as this was when the inlet velocity and inner pressure were being ramped in the first cycle. In Case 8, the L2 norm appeared to "converge" in the second cycle, similar to Case 9, where the 2nd and 3rd cycle norm was comparable to the 5th and 6h cycle norm. In Cases 3, 11, 12 and 16, true convergence was not achieved in six cycles, as it appeared that the norms were still decreasing. The high-frequency fluctuation in the norm of most cases is attributed to wall vibration, as the location of the wall varies randomly from cycle to cycle. Case 16, which had the highest level of variation in the L2 norm between cycles, stopped exhibiting visible differences in vibration amplitude (Figure 4a in the manuscript) in the 4th cycle. The amplitude of vibration in Case 16 was the most variable (Figure 4a in the manuscript), which is expected as the flow in this Case was known to not exhibit strong cycle-to-cycle convergence in a previous CFD study [Valen-Sendstad, 2011]. Ultimately, cycle 4 and onwards did not exhibit visible differences in the vibration amplitude or the spectrograms, so cycle 4 was analyzed in the current study.

Suppementary Figures

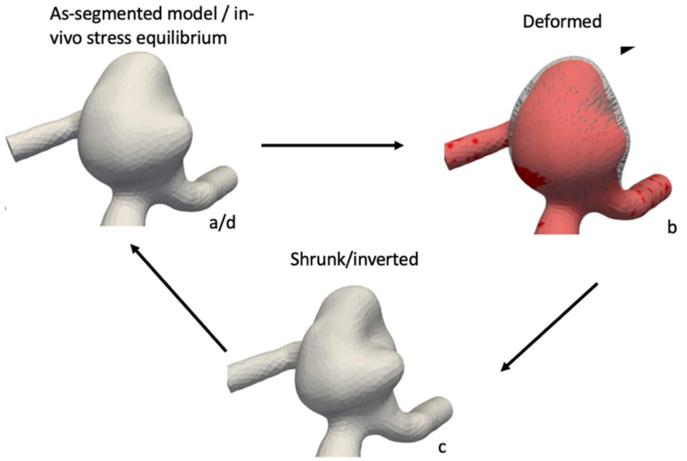

Supplementary Figure 1: Illustration of how we approximate the unknown in-vivo stress equilibrium between the blood pressure and stresses within the aneurysm wall at the time of image acquisition. We first apply a time-averaged perfusion pressure to the as-segmented model (panel a) and compute the deformation (panel b). The as-segmented model is then shrunk (or inverted) proportionally to the excessive deformation (panel b; grey) to obtain a zero-pressure and stress-

free geometry (panel c). This geometry is then gradually pressurized to closely approximate the as-segmented geometry (panel a/d), mimicking the in-vivo blood pressure and stress equilibrium. Note that panels b and c represent a pre-processing step.

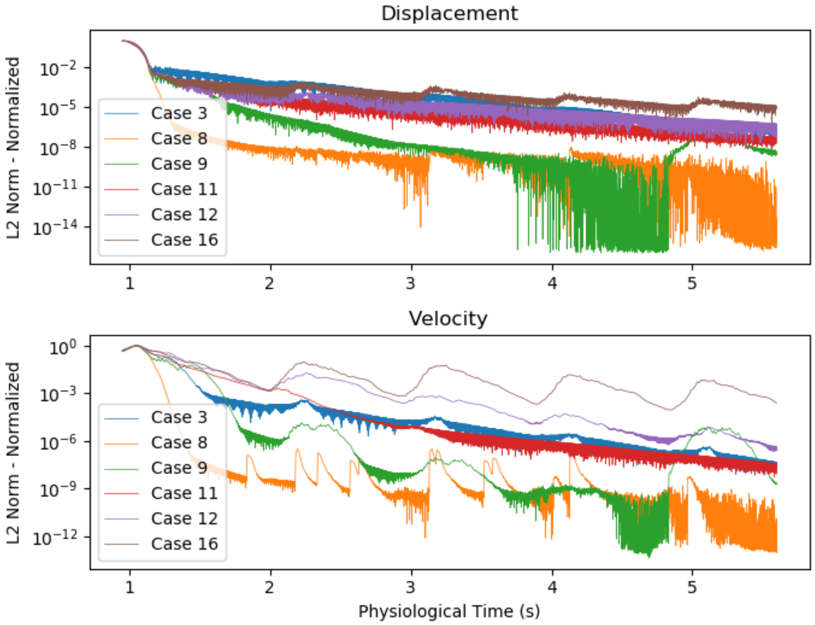

Supplementary Figure 2: Cycle-to-cycle convergence of wall displacement (top), fluid velocity (bottom)

Suppementary Reference

Valen-Sendstad, Kristian et al. 2011. "Direct Numerical Simulation of Transitional Flow in a Patient-Specific Intracranial Aneurysm." Journal of Biomechanics 44(16): 2826–32.
