# Supplementary material for: Understanding intracranial aneurysm sounds via high-fidelity fluid-structure-interaction modelling
Source: Commun Med (Lond). 2023 Nov 9;3:163. doi: 10.1038/s43856-023-00396-5 (PMC10636010; doi:10.1038/s43856-023-00396-5)
Supplement: Supplementary file 2 — Description of Additional Supplementary Files [file 43856_2023_396_MOESM2_ESM.pdf]

Description of Additional Supplementary Files

File Name: Supplementary Data 1
Description: Inlet flow rate and prescribed pressure – numerical data from Figure 1c**.**

File Name: Supplementary Data 2
Description: Displacement, velocity and spectrogram data for Figure 2.

File Name: Supplementary Data 3
Description: Modal amplitude data for Figure 3.

File Name: Supplementary Data 4
Description: Displacement and velocity amplitude data for each case for Figure 4.

File Name: Supplementary Data 5
Description: Displacement and velocity point traces for Figure 5**.**

File Name: Supplementary Data 6
Description: Point data for sac volume, aspect ratio, maximum flow instability amplitude and maximum flow velocity for Figure 6.

File Name: Supplementary Data 7
Description: Power spectra data for Figure 7.

File Name: Supplementary Video 1
Description: Real-time visualization of aneurysm flow and vibration for Case 3. Q-Criterion isosurfaces show the extent of high-frequency flow instabilities in purple (Q=5000 1/s) and (low frequency) stable vortex structures in light grey (Q=50,000 1/s). Displacement consists of only the >25 Hz vibration, amplified by a factor of 300 to make them visible. The same cycle is repeated 8 times. The point-wise spectrogram at the location of maximum amplitude is shown, with the fluid-velocity spectrogram displayed for the first 4 repetitions and the wall displacement spectrogram shown for the last 4 repetitions. The sound of the video is a direct sonification of the simulated velocity-time or displacement-time data from which the spectrogram was calculated. The sample points are indicated in red.

File Name: Supplementary Video 2
Description: Real-time visualization of aneurysm flow and vibration for Case 9. See caption of Supplementary Video 1 for details.

File Name: Supplementary Video 3
Description: Real-time visualization of aneurysm flow and vibration for Case 12. See caption of Supplementary Video 1 for details.

File Name: Supplementary Video 4
Description: Real-time visualization of aneurysm flow and vibration for Case 16. See caption of Supplementary Video 1 for details.

File Name: Supplementary Video 5
Description: Summary of mode shapes for Case 3, slowed by 100x. Note the depiction of the bruit, containing both expansion/contraction and random, rippling motions, compared to the consistent shapes of rocking modes 1-3 in each case. The mode shapes are not to scale and are normalized to the size of the sac so that each mode of a specific case is shown at the same visual amplitude. Top Right: Spectrogram of wall displacement, with the bruit shaded in yellow, and rocking modes of ascending frequency shaded in green, red and purple. Bottom Right: Spatial 99th percentile value of the RMS amplitude of vibration. The total vibration amplitude is displayed in black; the rocking modes displayed in green, red and purple; and the remaining bruit displayed in yellow.

File Name: Supplementary Video 6
Description: Summary of mode shapes for Case 9, slowed by 100x. See caption of Supplementary Video 5 for details.

File Name: Supplementary Video 7
Description: Summary of mode shapes for Case 12, slowed by 100x. See caption of Supplementary Video 5 for details.

File Name: Supplementary Video 8
Description: Summary of mode shapes for Case 16, slowed by 100x. See caption of Supplementary Video 5 for details.
